# Perceived Organizational Support and Proactive Innovation Behavior: The Mediating Role of Basic Psychological Needs

**DOI:** 10.3389/fpsyg.2022.804363

**Published:** 2022-03-18

**Authors:** Chuanhao Fan, Sijie Tang, Long Chen, Tingting Sun

**Affiliations:** ^1^Business School, Hohai University, Nanjing, China; ^2^Human Resources Research Institute, Ministry of Water Resources, Nanjing, China; ^3^Nanjing Hexi New Town Development and Construction Management Committee, Nanjing, China

**Keywords:** proactive innovation behavior, perceived organizational support, self-determination theory, autonomy need, competence need, relatedness need

## Abstract

Drawing upon self-determination theory, this study aimed to explore the mechanisms underlying the impact of perceived organizational support on proactive innovation behavior and reveal the serial mediation effects of basic psychological needs. We collected time-lagged data of 481 employees from research institutions in China, and structural equation modeling analyses were carried out to test the hypotheses. The results indicate that perceived organizational support is significantly and positively related to proactive innovation behavior, and this relationship was mediated by the need for autonomy, competence, and relatedness. These findings contribute new knowledge to proactive innovation behavior by providing a new perspective of the satisfaction of psychological needs. Theoretical and practical implications are discussed.

## Introduction

Focusing on the perniciousness of coronavirus disease 2019 (COVID-19), the pandemic and even further deterioration of the epidemic has brought uncertainty to future economic development (Rahmani and Mirmahaleh, 2020). As Huang and Li (2021) mentioned that “the global economy was estimated to have contracted 4.3 percent in 2020, which represents the deepest recession since World War II.” Supporting economic and social development cannot be separated from science and technology innovation which is the “primary driving force” in the post-epidemic era (Zhang et al., 2019). Therefore, contemporary organizations are facing the rapid rise of innovation pressure which has an important impact on their original management mode. Companies are compelled to think about how employees can spontaneously innovate methods and procedures at work (Parker, 2015). Given this widespread push for employees to take proactive innovation behavior at work (Khessina et al., 2018), management research should reveal contextual factors and process mediators that can promote employees’ proactive innovation behavior.

Based on the perspective of inner motivation for innovation of technical personnel, Zhao and other Chinese scholars (2014) define proactive innovation behavior as the behavior that employees voluntarily and sincerely prepare for future innovation and bravely face and solve problems in the process of innovation. Proactive innovation behavior is based on employees’ working resources and other conditions they master and cannot be separated from their working environment (Chen et al., 2017). Perceived organizational support means that the organization attaches importance to the benefits and contributions of employees, supports employees, and fully considers their needs (Luksyte and Spitzmueller, 2015). Perceived organizational support plays a crucial role in the employee–employer relationship and has a significant impact on employees’ commitment, satisfaction, and other positive behaviors (Panaccio and Vandenberghe, 2009; Kurtessis et al., 2017). When employees perceive the support from the organization, they will have the intention of returning (Dai et al., 2018), while doing well in-role behavior, they will further show proactive innovation behavior.

Most researchers agree that innovation is related to the generation, adoption, or implementation of creative ideas (Kanter, 1988). However, innovation behavior is often acknowledged as a single dimension composed of progressive stages of behaviors by previous studies (e.g., Jong and Hartog, 2010). Fortunately, some researchers have begun to focus on differences within innovation behavior. For example, Veenendaal and Bondarouk (2019) determined how human resource management promotes three different types of innovation behavior related to idea generation, idea championing, and idea application. Proactive innovation behavior consists of three dimensions: spontaneity, previous preparation, and cross-obstacles (Zhao et al., 2014), and previous preparation and cross-obstacles correspond to different dimensions of idea generation and idea implementation, respectively. In addition, innovation behavior starts with the previous preparation, and an individual seeks sponsorship and cross-obstacles during the next stage of the process (Scott and Bruce, 1994). At present, it is still controversial whether the generation of creative ideas can lead to the implementation of those ideas (Veenendaal and Bondarouk, 2019). Based on the above considerations, this article considers three variables of proactive innovation behavior separately and explores under what conditions can the idea generation be transformed into idea implementation.

We adopted self-determination theory to analyze the theoretical conceptual framework. Self-determination theory suggests that creating a work environment in which the employee feels supported can lead to an individual’s autonomous motivation and then enhance better job performance, especially on exploratory activities (Deci and Ryan, 2000). In other words, organizational factors have an impact on promoting motivation and behavior through the mediating role of basic psychological needs. In addition, supporting and satisfying three basic psychological needs are highly correlated at a general level because when employees have a sense of autonomy, they attempt to meet the other needs (Deci et al., 2017). Some studies have found that people with high autonomy orientation feel more competent and stay in more contact with their colleagues (e.g., Baard et al., 2004). Specifically, Kluwer et al. (2019) proved that the needs for autonomy and relatedness interact with each other and further affect accommodation together. Additionally, autonomy need is directly related to competence need in Ruth’s study (2020). Consequently, we propose and examine the indirect effect of perceived organizational support on the level of competence and relatedness need through autonomy need.

The significance of this article mainly includes three points. First, it expands how the satisfaction of psychological needs plays a mediating role between perceived organizational support and proactive innovation behavior. Different from the passive innovation behavior caused by performance pressure, proactive innovation behavior is generated by the internal motivation of employees (Kleysen and Street, 2001). Therefore, environmental factors may only be the distal influencing factor of proactive innovation behavior, whereas individual motivation is the proximal influencing factor of proactive innovation behavior (Vansteenkiste and Ryan, 2013). Second, researchers often treat the needs for autonomy, competence, and relatedness as the independent determinants or as a merged construction, less is known about the connection of three types of basic psychological needs (Kluwer et al., 2019). We verified that relatedness need and competence need may be influenced by autonomy need. Third, this article attempts to call for the dispute that the generation of novel ideas may not necessarily result in the adoption or implementation of those ideas. To sum up, this study proposes the chain mediation hypothesis of basic psychological needs to comprehensively analyze the relationship between perceived organizational support and proactive innovation behavior.

## Theory and Hypotheses

### Self-Determination Theory

In this study, self-determination theory provides a theoretical basis for explaining the generation and influence of individual motivation and behavior. The core of this theory is that an individual has three types of basic psychological needs, namely, autonomy need, competence need, and relatedness need (Deci et al., 1989). Autonomy need is recognized that individuals make choices and decisions according to their subjective intentions rather than external forces (Deci et al., 1989). Competence need refers to employees feeling like they are capable and confident in completing challenging job tasks and achieving ideal results (Van den Broeck et al., 2010). The satisfaction of relatedness need requires a sense of belonging, caring, and being an important and contributing member of a group (Baumeister and Leary, 1995). The satisfaction of three basic psychological needs will promote high-quality, sustainable motivation, and experience wellbeing (Vansteenkiste et al., 2020).

In addition to systematizing basic psychological needs, self-determination theory also distinguishes two kinds of motivation and posits that an individual’s different cognition of the environment will produce different types of motivation (Ryan, 1982; Battaglio et al., 2021). According to the cognitive evaluation theory, the cognition of external events can be divided into informational cognition and controlling cognition (Bandura, 1991). Informational cognition refers to an interpretation of the external environment as a kind of support. For example, an organization provides the opportunity for employees to make independent decisions, which can stimulate individuals’ autonomous motivation and they are more likely to perform better and learn better (Deci et al., 2017). Controlling cognition refers to that individual interprets the external environment as a constraint or compulsion. For example, strict performance standards result in the controlled motivation of employees, which have negative spillover effects on subsequent performance (Kleysen and Street, 2001).

### Research Hypotheses

Under the special cultural background of China, the innovation behavior of employees may not be out of their own will but subordinated to the organizational system or authoritative instructions (Ma and Wang, 2016). Zhao et al. (2014) distinguished proactive and passive innovation behaviors based on the Chinese context and defined proactive innovation behavior as the behavior that employees voluntarily prepare for future innovation and bravely face and solve problems in the process of innovation which is motivated by inner will (Zhao and Han, 2016). Proactive innovation behavior has three characteristics: spontaneity, previous preparation, and cross-obstacles. Specifically, previous preparation refers to the preparation made in advance by an individual to put forward and implement innovative ideas that include thinking preparation and resource preparation. The complexity of innovation requires an individual to have a strong ability to bear difficulties, a firm determination to overcome difficulties and show perseverance in the face of innovation failure that is called cross-obstacles. Spontaneity refers to an individual’s desire to act automatically without any external force. Spontaneity is the most critical characteristic of proactive innovation behavior, and it is the behavior that employees do not need to be “informed” (Strauss et al., 2015).

#### Perceived Organizational Support and Previous Preparation

Eisenberger et al. (1986) defined perceived organizational support as employees perceiving that their organizations attach importance to their benefits and contributions and fully considering their interests and needs. Perceived organizational support emphasizes commitment, rewarding, and appreciating employees and inspiring them to come up with novel ideas (Neves and Eisenberger, 2014). We can understand perceived organizational support through the norm of reciprocity (Deconinck, 2010). That is, when the organization gives support to employees generously, employees will feel this support and will be willing to show positive behaviors to respond to this support (James et al., 2015). This study believes that perceived organizational support can directly stimulate previous preparation for two reasons. On the one hand, perceived organizational support can be defined as the total amount of support from colleagues and superiors that is believed to help employees perform their job responsibilities successfully (Luthans et al., 2008). According to self-determination theory, this non-controlling information helps employees to be prepared for thinking (Vansteenkiste and Ryan, 2013); i.e., employees can consult their superiors and colleagues about the difficulties they encounter in the future innovation process and put forward more novel and practical innovative ideas after understanding various perspectives. On the other hand, China has issued policies to support and encourage innovation and entrepreneurship in universities, research institutes, and other public institutions, which provided resources in terms of intellectual property management, post-management, and construction of sharing platforms for employees to support their innovation. In view of the job demands-resources model, job resources can help employees treat with job demands, improve the learning and development of qualified employees, induce potential work motivation (Gillet et al., 2017), and stimulate excellent work performance such as innovation behavior (Chen et al., 2017). Therefore, the following hypothesis is proposed:

Hypothesis 1: Perceived organizational support is positively associated with previous preparation.

#### The Mediating Role of Basic Psychological Needs Between Perceived Organizational Support and Previous Preparation

In light of self-determination theory, basic psychological needs are psychological nutrients to an individual, and it is believed that some environmental factors may promote the fulfillment of one need rather than the other (Deci et al., 2001). The need for competence is widely regarded as the core element of internal motivation (Deci et al., 2017). When an organization perceives employees’ contributions and supports them to do challenging work, the need for competence will be satisfied to a higher degree (Yan and Fan, 2020). Feelings of competence will stimulate the motivation for learning, improving skills, and other behaviors, which enables them to complete job tasks beyond their current capabilities (Austin and Costabile, 2021). That is, employees will be able to devote more energy to identify problems and make more preparations that are intrinsic in nature. In addition, competent employees who win support from the organization may see the organization as a place to further develop their knowledge and skills (Chong et al., 2020), and then, they are easy to produce a strong achievement motivation and do more early-stage preparations for future proactive innovation behavior (Men et al., 2015). Therefore, the following hypothesis is proposed:

Hypothesis 2a: Perceived organizational support improves previous preparation by enhancing the competence need.

Supporting and satisfying three basic psychological needs are highly correlated at a general level because when employees have a sense of autonomy, they attempt to meet the other need on their own (Deci et al., 2017). It is believed that psychological needs may interact with each other (Deci and Ryan, 2000). In the process of satisfying basic psychological needs, autonomy need is often the first to be satisfied (Liu et al., 2011). Supportive organizations tend to create a relaxed working atmosphere and flexible working environment to support employees’ innovation (Jing et al., 2011). In this process, employees who get more autonomous opportunities at work will make efforts to exercise and express abilities, which bring a sense of control in their actions and promote the satisfaction of competence needs (Zhou and Bao, 2005). The competence need can also promote employees to have the confidence to find new problems in work and put forward new methods (Austin and Costabile, 2021), so as to make thinking preparation for innovation. Relevant empirical evidence also confirmed this view. Wielenga-Meijer et al. (2010) show that the satisfaction of autonomy need could stimulate employees’ potential, satisfy their competence need and, thus, promote their exploration and innovation behavior. Therefore, the following hypothesis is proposed:

Hypothesis 2b: Perceived organizational support enhances the competence need by enhancing the autonomy need and finally improves previous preparation.

Although an individual’s satisfaction of autonomy need has been proved to be a predictive factor of employee initiative behaviors (Yu and Davis, 2016), the satisfaction of autonomy need has not been shown to be a proximal factor of proactive innovation behavior. The satisfaction of autonomy need can improve the relationship energy between managers and subordinates (Deci et al., 2001); in other words, autonomy need can promote the satisfaction of relatedness need. Consequently, autonomy support is expected to facilitate experienced satisfaction of the need for relatedness, as was found in Baard et al. (2004)’s study. Employees who have a high-level relatedness satisfaction are more likely to obtain resources and valuable information from others (Yimo et al., 2019) and integrate the views of interdependent members to attain a higher innovation performance (Li and Liao, 2014). Thus, the more information resources the employees have the more thinking preparation for proactive innovation behavior they will make. Existing research has also confirmed that autonomy need and relatedness need interact with each other and further affect accommodation together (Kluwer et al., 2019). Therefore, the following hypothesis is proposed:

Hypothesis 2c: Perceived organizational support enhances the relatedness need by enhancing the autonomy need and finally improves previous preparation.

#### Perceived Organizational Support and Cross-Obstacles

Due to the complexity and uncertainty of innovation, employees are required to have a strong ability to withstand difficulties and a firm determination to overcome them (Luthans et al., 2008). As mentioned above, employees whose autonomy need has been satisfied are more likely to participate in challenging work, increase the possibility and confidence of completing work tasks, and then bring a high sense of competence. An employee who has the satisfaction of competence need will have a strong tolerance, strong determination to overcome difficulties, and resolute perseverance in the face of innovation failure (Zhao et al., 2014). Only in this way, innovation can achieve the desired results in repeated failure and attempts. In addition, the competence need can promote the generation of employees’ achievement motivation. Employees who have high achievement tend to take on more challenging and high-goal jobs (Schoen, 2015), invest a lot of time and energy to enhance their motivation, and look forward to a sense of accomplishment after completing tasks (LePine et al., 2004). Therefore, the following hypothesis is proposed:

Hypothesis 3a: Perceived organizational support enhances the competence need by enhancing the autonomy need and finally improves cross-obstacles.

From the perspective of process-oriented, innovation behavior is summarized as a series of changes that individual generates innovative ideas and put them into the innovative application through efforts (Wang and Yang, 2017). Proactive innovation behavior starts with the generation of ideas but the generation of ideas may do not guarantee that the novel ideas will be put into practice, because creative ideas are often characterized by high risk and uncertainty, which may be contrary to personal preferences and reality (West, 2002; Baer, 2012). More organizational support results in a reciprocal environment in which organizations generously support employees and induce them to show higher levels of proactive innovation behaviors to respond to the support. Specifically, the more adequate work resources and thinking preparation, the more determined and tenacious employees will be to overcome difficulties in the process of innovation (Gagné, 2003). Hence, it can be seen that innovation behavior is a process of leap development from quantitative changes to qualitative changes. Therefore, the following hypothesis is proposed:

Hypothesis 3b: Perceived organizational support improves cross-obstacles by enhancing previous preparation.

#### Perceived Organizational Support and Spontaneity

In the atmosphere of high-level organizational support, employees are encouraged to make their own decisions and take creative actions, which is the spontaneous behavior of pleasure and interest (Deci et al., 2017; Tsachouridi and Nikandrou, 2018). However, this involvement and commitment to challenging and creative activities depend on the degree to which employees are satisfied with basic psychological needs (Deci and Ryan, 2000). As mentioned above, perceived organizational support will affect relatedness need through the satisfaction of autonomy need. Because of the interdependent nature of teamwork (Li et al., 2018), when the relatedness need is satisfied, an employee will be more likely to have positive interactions with others and access valuable information in the enterprises (Yimo et al., 2019). In this way, employees’ interest and curiosity in innovation can be stimulated and they will be more eager to solve problems in a novel and ingenious way, which is conducive to the generation of the intrinsic motivation of proactive innovation behavior. The intrinsic motivation generated by satisfying basic psychological needs is the lasting power to promote proactive innovation behavior (Yan and Fan, 2020). Therefore, the following hypothesis is proposed:

Hypothesis 4: Perceived organizational support enhances the relatedness need by enhancing the autonomy need and finally improves spontaneity.

**FIGURE 1:**
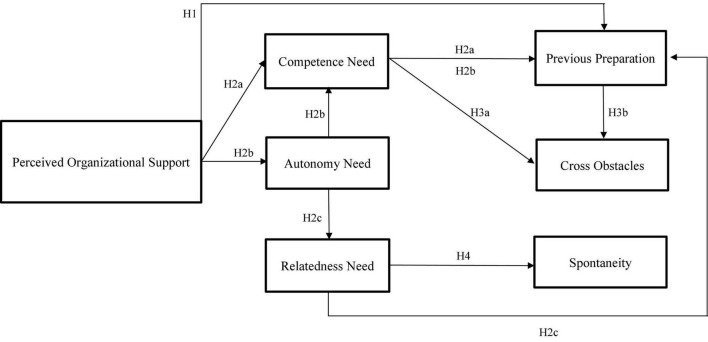
Theoretical model.

Our theoretical model is shown in Figure 1.

## Materials and Methods

### Sample and Procedures

We collected data from 19 research institutes in nine cities (e.g., Wuhan, Beijing, and Nanjing). With the help of heads of HR departments of each organization, we drew employees randomly and invited them to complete the questionnaire. Considering these organizations in different cities, we used an online questionnaire to speed up the survey. The heads of the HR departments sent the participants the link to the questionnaire and reminded those who failed to respond in the given time. All the participants were informed that the data collected would only be for academic purposes and incur no negative impact. Also, their individual information would not be shared with the organization they served. The data for the study were collected through employee self-reporting in three waves. The first-round questionnaire survey was conducted in May 2020 (T1), which included questions about demographic information, perceived organizational support. All the questionnaires were coded to enable us to match them with the questionnaires we would collect during the second and third rounds of data collection. The second-round questionnaire survey was conducted in July 2020 (T2), which included questions about demographic information, basic psychological needs. The third-round questionnaire survey was conducted in September 2020 (T3), which included questions about demographic information, proactive innovation behavior.

In total, 600 questionnaires were sent out and 481 were matched successfully. The recovery rate of effective questionnaires was 87.77%. Among the final sample, 64% of the respondents were men, and 36% were women. Those aged distributed in the 1980s and 1970s were the most, which accounts for 46.1 and 22.5%, respectively. In terms of educational background, the majority of respondents received master’s degree, bachelor’s degree, and doctor’s degree, which accounts for 43.7, 31.2, and 20.8%, respectively. The position was divided into four levels: division level, section level, section member, and others, which accounts for 7.5, 23.7, 29.3, and 39.5%, respectively. As for the title, 40.1% of the respondents received the vice-senior title, 31.2% received the middle title, 18.7% received the primary title, and 10% received the senior title.

### Measures

The measurement tools used in this study were based on the mature scale studied by predecessors. Through semistructured interviews with employees and experts in several research institutes, the content of the scale items was modified and improved to form the final questionnaire. A 7-point Likert scale was used for its scoring (from 1 = strongly disagree to 7 = strongly agree).

#### Perceived Organizational Support

We used Rogg et al. (2001) 10 items to measure perceived organizational support. A sample item is “Managers consistently treat everyone with respect.” These items came from two factors of supportive organizational climate, measuring managerial competence, and cooperation or coordination, respectively. This shortened scale has been used by Luthans et al. (2008) and showed validity. As far as this study is concerned, it contains the key construct relevant to the satisfaction of employees’ basic psychological needs. The Cronbach’s alpha for this scale in this study was 0.934.

#### Basic Psychological Needs

We measured basic psychological needs by revising the initial measurement developed by Gagné (2003). The revised scale consists of 17 items: the autonomy need scale has five items (e.g., “You can decide how you work”); the competence need scale has eight items (e.g., “The exchange opportunities provided by your organization have promoted your career development”); the relatedness need scale has four items (e.g., “You enjoy providing information to others”). The Cronbach’s alpha coefficients in this study were 0.925, 0.910, and 0.852 for autonomy need, competence need, and relatedness need, respectively.

#### Proactive Innovation Behavior

The 12-item scale developed by Zhao et al. (2014) was used to measure proactive innovation behavior. A sample item was “You genuinely want to make a contribution to your organization.” The Cronbach’s alpha coefficients were 0.929, 0.940, and 0.882 for spontaneity, previous preparation, and cross-obstacles, respectively.

Control variables: We classify demographic variables such as gender, age, and educational levels as control variables in this study because previous studies indicated that such variables were potentially related to an individual’s innovation behavior (Zhang and Bartol, 2010; Ng and Feldman, 2013). All demographic variables were given the dummy codes.

### Data Analysis

Before sending the data for hypothesis testing, we checked the discriminant validity among the multi-item constructs and common method variance to assess the measurement model. Pearson’s correlation analyses were carried out to lay a foundation for hypotheses testing. The hypotheses were tested using structural equation modeling. Furthermore, we ran an additional analysis on whether the results were consistent across certain demographic groups.

## Results

### Common Method Variance

Since the variables measured were all from the self-reported data of employees, there may be the problem of common method variance. Harman single factor test method was adopted to test the common method variance. Under the condition of no factor rotation, the variance contribution rate of the first factor precipitated was 41.477%, lower than the critical value of 50% (Podsakoff and Organ, 1986), which indicates that the common method variance was within the acceptable range in this study.

### Discriminant Validity

We used AMOS 21.0 software to perform confirmatory factor analysis. The seven-factor model fit indexes (χ^2^ = 2,419.69, *df* = 681, TLI = 0.895, CFI = 0.904, RMSEA = 0.073) were better than other models shown in Table 1, which indicates that all variables in the theoretical model had good discriminant validity. Meanwhile, the standardized factor load of all items in the seven-factor model was greater than 0.7, further providing support for the convergent validity of the seven variables.

**TABLE 1:** Results of confirmatory factor analysis.

Model	Model combination	χ^2^	df	χ^2^/df	RMSEA	NFI	TLI	CFI
One-factor model	POS + RE + AU + COM + SP + PP + CO	9904.098	702	14.108	0.165	0.449	0.463	0.466
Two-factor model	POS + RE + AU + COM; SP + PP + CO	9044.894	701	12.903	0.157	0.52	0.513	0.539
Three-factor model	POS; RE + AU + COM; SP + PP + CO	8180.788	699	11.704	0.149	0.566	0.562	0.587
Four-factor model	POS; RE + AU + COM; SP + PP; CO	5196.814	696	7.467	0.116	0.724	0.735	0.751
Five-factor model	POS; RE; AU; COM; SP + PP + CO	4609.773	692	6.662	0.109	0.718	0.732	0.749
Six-factor model	POS; AU; RE + COM; SP; PP; CO	3336.693	687	4.857	0.090	0.823	0.842	0.854
Seven-factor model	POS; RE; AU; COM; SP; PP; CO	2419.688	681	3.553	0.073	0.872	0.895	0.904

*POS, perceived organizational support; AU, the need for autonomy; COM, the need for competence; RE, the need for relatedness; SP, spontaneity; PP, previous preparation, CO, cross-obstacles; + represents two factors to synthesize a variable. The abbreviations of variables are the same as below.*

This study further analyzed the extracted mean variance values of seven variables, and the arithmetic square root of the extracted mean variance values were shown in bold in Table 2. The extracted mean variance values of all variables were greater than 0.5, which further indicated that the seven variables in this study all had good convergence validity. At the same time, the arithmetic square root of the extracted mean variance values of the seven variables was all greater than the correlation coefficient between these variables and other variables, which once again verified the good discriminative validity between the core constructs in this study.

**TABLE 2:** Means, standard deviations, and correlations of variables.

Variables	1	2	3	4	5	6	7	8	9	10	11	12
1. Gender												
2. Age	–0.042											
3. Education	−0.133**	0.221**										
4. Position	–0.074	0.360**	0.006									
5. Title	–0.079	0.653**	0.216**	0.402**								
6. POS	0.055	–0.048	0.044	0.133**	–0.089	**(0.797)**						
7. RE	0.040	0.141**	–0.016	0.192**	0.115*	0.199***	**(0.775)**					
8. AU	0.071	0.005	0.009	0.196**	0.003	0.551***	0.000	**(0.844)**				
9. COM	–0.002	0.027	0.005	0.226**	0.019	0.482***	0.000	0.000	**(0.750)**			
10. SP	0.020	0.099*	0.154**	0.119**	0.189**	0.109*	0.484***	-0.030	0.035	**(0.872)**		
11. PP	0.054	0.025	0.019	0.184**	–0.001	0.697***	0.198***	0.519***	0.453***	0.000	**(0.775)**	
12. CO	–0.024	0.010	0.009	0.042	–0.021	0.027	0.055	0.033	0.036	0.000	0.000	**(0.932)**
M	0.360	3.780	2.809	2.414	2.992	4.112	5.264	3.741	4.020	5.692	4.414	4.321
SD	0.480	1.313	0.812	0.904	0.966	1.143	1.027	1.348	1.194	1.090	1.215	1.386

*n = 481.*

*Numbers in parentheses on the diagonal are reliabilities of these variables.*

*indicates p < 0.05, **indicates p < 0.01, ***indicates p < 0.001.

We performed a supplementary verification of discriminant validity. A novel approach for assessing discriminant validity was proposed by Henseler et al. (2015), that is the heterotrait–monotrait (HTMT) ratio of correlations. A threshold of less than 0.85 can be considered to reliably distinguish between those pairs of latent variables. The HTMT ratio results are shown in Table 3. All ratios were less than 0.8, which indicated good discriminative validity.

**TABLE 3:** Results of heterotrait–monotrait (HTMT) ratio.

HTMT	POS	RE	COM	AU	SP	PP	CO
POS	–						
RE	0.461	–					
COM	0.737	0.651	–				
AU	0.761	0.449	0.763	–			
SP	0.234	0.589	0.285	0.182	–		
PP	0.771	0.531	0.746	0.747	0.348	–	
CO	0.124	0.155	0.160	0.145	0.144	0.229	–

### Correlation Analysis

Table 2 presents the descriptive statistics and correlations. At a significant level of 0.05, perceived organizational support was significantly correlated with spontaneity, previous preparation, and cross-obstacles. The hypotheses were verified preliminary, which laid a foundation for hypotheses testing in the following article.

### Hypothesis Testing

**FIGURE 2:**
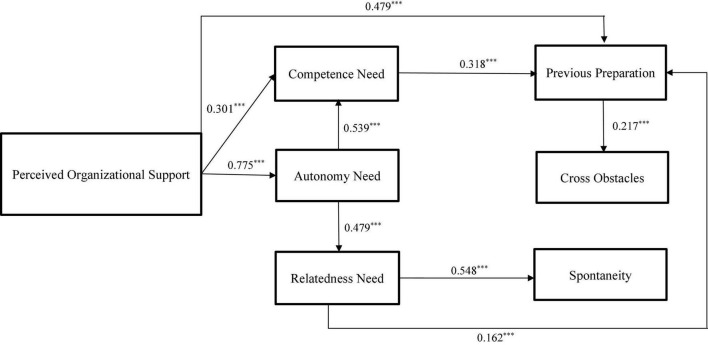
Results of the structural equation. ***indicates *p* < 0.001.

The structural equation model was used to test the hypotheses in this study, and all indexes met acceptable standards (χ^2^/*df* = 3.721, NFI = 0.880, TLI = 0.871, CFI = 0.879, RMSEA = 0.075). The standardized fitting results are shown in Figure 2. Perceived organizational support was significantly and positively related to previous preparation (β = 0.479, *p* < 0.01). Hypothesis 1 was supported.

Table 4 represents the results of the structural equation through the bootstrapping test method proposed by Taylor et al. (2008). The results of mediation analyses, concerning Hypothesis 2a, showed that the need for competence was a mediator in the relationship between perceived organizational support and previous preparation (β = 0.082, [95% CI: 0.047–0.136]). Therefore, Hypothesis 2a was supported. The chain mediating effect value of the need for autonomy and competence between perceived organizational support and previous preparation was 0.114 ([95% CI: 0.065–0.173]), which indicates that the mediating effect was significant. Therefore, Hypothesis 2b was supported. The chain mediating effect value of the need for autonomy and relatedness between perceived organizational support and previous preparation was 0.052 ([95% CI: 0.026–0.082]). The mediating effect was significant. Therefore, Hypothesis 2c was supported. The chain mediating effect value of the need for autonomy and competence between perceived organizational support and cross-obstacles was −0.007 ([95%CI: −0.061 to 0.040]), which includes 0. The mediating effect was not significant. Therefore, Hypothesis 3a was not supported. The value of the indirect effect of perceived organizational support on cross-obstacles through previous preparation was 0.111 ([95%CI: 0.061–0.175]). Therefore, Hypothesis 3b was supported. The chain mediating effect value of the need for autonomy and relatedness between perceived organizational support and spontaneity was 0.163 ([95%CI: 0.122– 0.220]). The mediating effect is significant. Therefore, Hypothesis 4 was supported.

**TABLE 4:** Bootstrap test for mediating effect.

Paths	Coefficient (β)	Bias-corrected		Results
		95%CI		
		Lower	Upper	
POS → PP	0.475	0.708	0.833	H1 supported
POS → COM → PP	0.082	0.047	0.136	H2a supported
POS → AU → COM → PP	0.114	0.065	0.173	H2b supported
POS → AU → RE → PP	0.052	0.026	0.082	H2c supported
POS → AU → RE → CO	−0.007	−0.061	0.040	H3a not supported
POS → PP → CO	0.111	0.061	0.175	H3b supported
POS → AU → RE → SP	0.163	0.122	0.220	H4 supported

### Additional Analyses

Because of the significant correlation between position and other constructs, our study used the multi-group analysis method proposed by Chin (2004) to examine the hypothesis on the moderating role of position. We divided the position into two groups: chief staff member and above, below of the chief staff member. First, the measurement invariance was tested and the results showed that the measurement model was invariant between different groups (Δχ^2^ = 24.181, Δ*df* = 32, *p* = 0.838 > 0.05). Subsequently, by setting the path coefficients to be the same, we compared Δχ^2^ between the unconstrained model and the constrained model. The goodness-of-fit of the unconstrained model was good (χ^2^/*df* = 2.603, RMSEA = 0.058, CFI = 0.860); the goodness-of-fit of the constrained model was good too (χ^2^/*df* = 2.550, RMSEA = 0.057, CFI = 0.860). There was no significant difference between the unconstrained model and the constrained model (Δχ^2^ = 33.338, Δ*df* = 42, *p* = 0.827 > 0.05), which indicates that position did not play a moderating role in our construct model. Next, we calculated the coefficients and *t*-values of the hypothesized paths to evaluate the significance of the relationships in each group. The results shown in Table 5 indicated that different groups had significant differences in the relationship between perceived organizational support and autonomy need. There was no statistically significant difference in other paths.

**TABLE 5:** Results of multi-group analysis for position group.

Path	Section level		Below of		Critical ratios of differences
	and above		section level		
	Standardized path coefficient	*t*-value	Standardized path coefficient	*t*-value	
POS → AU	0.787***	11.438	0.764***	14.451	1.849*
POS → COM	0.175	1.550	0.372***	5.657	1.249
AU → RE	0.516***	6.143	0.437***	7.334	0.007
AU → COM	0.582***	4.937	0.498***	7.217	0.601
POS → PP	0.377***	4.270	0.533***	7.781	0.242
COM → PP	0.350***	3.990	0.287***	4.308	1.129
RE → PP	0.222***	2.998	0.141***	3.254	1.151
RE → SP	0.589***	7.184	0.512***	8.831	0.076
PP → CO	0.076	0.595	0.297***	2.97	0.914
COM → CO	0.083	0.661	−0.064	−0.671	0.931

*Indicates p < 0.05, ***indicates p < 0.001.

## Discussion

Drawing from self-determination theory, this study was conducted with 481 employees of research institutions in China to understand the impact of perceived organizational support on proactive innovation behavior through employee basic psychological needs. The results suggest the following: (1) perceived organizational support has a directly and significantly positive effect on previous preparation; (2) perceived organizational support is positively associated with previous preparation through the need for competence; perceived organizational support affects competence need and relatedness need through the satisfaction of autonomy need and finally affects previous preparation; (3) perceived organizational support is positively associated with cross-obstacles through previous preparation; and (4) perceived organizational support affects relatedness need through the satisfaction of autonomy need and finally affects spontaneity. This study has made several important contributions both in theory and in practice.

### Theoretical Implications

An important theoretical implication is that perceived organizational support plays a crucial role in proactive innovation behavior, which triggers a motivation-promoting process by satisfying psychological needs. Under a high level of organizational support atmosphere, individuals will have a certain sense of obligation and be willing to turn it into positive innovation behavior to reward the organization (Dai et al., 2018), which is consistent with the results of recent relevant studies. For instance, Paulsen et al. (2013) found that perceived organizational support significantly and positively affects employees’ innovation behavior. Although previous studies have demonstrated that perceived organizational support is one of the predictors of an individual’s creativity (e.g., Diliello et al., 2011; Ibrahim et al., 2016; Duan et al., 2020), few studies have focused on how basic psychological needs affect the relationship between them. As Bäckström and Bengtsson (2019) mentioned that researchers are supposed to pay more attention to investigating “how organizational support for innovative behavior can generate, develop, and implement ideas?” This study extends the boundary condition of self-determination theory and opens the black box that perceived organizational support acts on the proactive innovation behavior by satisfying the basic psychological needs. This insight holds important implications for promoting employees’ proactive innovation behavior.

Second, our results show that the satisfaction of three psychological needs not only be used as a composite (Deci et al., 2017) but also has a chain mediation in the relationship between perceived organizational support and proactive innovation behavior. Although other studies have explored the mediation effect of basic psychological needs (e.g., Amato et al., 2016; Lin and Chan, 2020), to our knowledge, few studies have examined the connection of three dimensions of basic psychological needs. Similar to Kluwer et al. (2019) and Wong (2020), it is easier to find the direct relationship between autonomy need, competence need, and relatedness need. Specifically, our study extends the current research by verifying that satisfying three basic psychological needs is highly correlated at a general level, and the need for autonomy as often the first need to be satisfied has an effect on both competence need and relatedness need, which provides a more comprehensive model to understand the psychological process of individual’s motivation and behavior.

Innovation is never accomplished overnight because of its complexity, uncertainty, and fuzziness. Throughout history, innovation behavior is often characterized as a series of changes that individual generates novel ideas and put them into innovative application (Wang and Yang, 2017). At present, the conceptualization of the innovation process mainly focuses on the classification of innovation activities (e.g., incremental innovation and disruptive innovation), but there is little research on how innovation behavior is sequenced and spaced (Wang and Dass, 2017). The generation of novel and creative ideas that can lead to the implementation of those ideas is still controversial. We call for this dispute by demonstrating the fact that previous preparation mediates the relationship between perceived organizational support and cross-obstacles. Additionally, we propose that innovation behavior is a process of leap development from quantitative changes to qualitative changes. This finding is consistent with our interviews’ results.

However, it should be noted that the chain-mediating effect value of the need for autonomy and competence between perceived organizational support and cross-obstacles is not significant. For some employees, being in a supportive atmosphere will have high achievement through the satisfaction of competence to carry out challenging work (Skerlavaj et al., 2017). However, the perception of competence is fragile under some circumstances which may result in employees’ negative behaviors, such as reducing effort or giving up in the process of innovation (Nerstad et al., 2020). In response to this conclusion, some researchers explain that environmental conditions may affect the strength of external feedback forced on an individual’s cognition and behaviors (Bandura, 1991). Therefore, the explanation of the contradictory finding requires more discussion on individual differences such as personality, traits, and cognition.

### Practical Implications

At present, many employees in Chinese enterprises have to carry out “innovative behavior” under organizational pressure. Such innovative behavior is passive and coping, and it is difficult to bring long-term performance to the organization. How to stimulate employees’ proactive innovation behavior is an urgent topic to discuss in the post-epidemic era. Based on the results of this article, we offer the following management advice to organizations and leaders.

First of all, the results clarify the relationship between the generation of novel ideas and the implementation of those ideas and put forward the importance of organizational support in promoting the implementation of ideas. Lacking resources, employees’ proactive innovation behavior is just like “cooking without rice” as the Chinese saying goes. Only by providing employees with good innovation resources such as funds, equipment, and technology, and a comfortable and fair working environment can be the proactive innovation behavior be carried out smoothly. Therefore, organizations should strengthen relevant supporting policies and measures, set up a perfect innovation service support system, and give employees learning resources support, and so on, to provide more thinking and resource preparation for proactive innovation behavior.

Second, the results indicate that basic psychological needs play a crucial role in the relationship between perceived organizational support and proactive innovation behavior. By understanding the differences in individuals’ psychological needs, organizations will be more likely to achieve the desired results. On the one hand, leaders should increase the degree of authorization, give employees the right to adjust the way they work, and give employees full autonomy and emotional support. On the other hand, providing interesting job tasks is characterized as various, challenging and meaningful for employees which can satisfy the competence need. It should also provide a certain growth space and development platform for employees to display their internal initiative and autonomy to a greater extent.

Finally, organizations should promote employees’ sense of belonging to the organization by creating a good team atmosphere, which can build a bridge to enhance employees’ proactive innovation behavior. The establishment of different levels of interpersonal belonging requires the support of leaders, colleagues, and subordinates. Therefore, organizations should create an atmosphere of teamwork, such as providing platforms for interaction and communication with colleagues at different levels, which allows different perspectives and constructive dialogue among team members and enhances employees’ sense of belonging to the organization.

## Limitations and Future Research

Several limitations need to be taken into account in interpreting the current findings. First, the dataset was collected from 19 research institutions in China, which limits its generalizability. Future research should replicate our results in other cultural backgrounds to generalize the findings. Second, it was likely to cause common method biases because our study was collected through self-report scales. Therefore, the perspectives of supervisors and peers should be used to examine employees’ proactive innovation behavior. In addition, multilevel studies are supposed to be conducted in the future because our analysis was performed at the individual level. Finally, whereas this study focused solely on the role of basic psychological needs in the relationship between perceived organizational support and proactive innovation behavior, our findings need to be complemented by studies that additionally investigate boundary conditions. In this regard, job characteristics (Cerne et al., 2017), high-performance work systems (Chiang et al., 2015), and individual differences (Bunce and West, 1995) may have different effects on this mechanism. Therefore, further research can be done to examine the moderating role of other factors through which perceived organizational support promotes proactive innovation behavior.

## Conclusion

We developed a more comprehensive model of how perceived organizational support affects proactive innovation behavior through the satisfaction of basic psychological needs in the Chinese context. In light of self-determination theory, we demonstrated the chain mediating effect of basic psychological needs in the relationship between perceived organizational support and proactive innovation behavior. Furthermore, we demonstrate that the generation of novel ideas in a supportive organizational atmosphere can facilitate their implementation. In total, our research thereby contributes to the proactive innovation behavior literature and psychological needs literature.

## Data Availability Statement

The original contributions presented in the study are included in the article/supplementary material, further inquiries can be directed to the corresponding author.

## Author Contributions

LC and ST contributed to the conception and design of the study, and responsible for reviewing and editing sections of the draft. CF and TS conducted questionnaire design and statistical analysis, and wrote the original draft. All the authors contributed to manuscript revision, and read and approved the submitted version.

## Conflict of Interest

The authors declare that the research was conducted in the absence of any commercial or financial relationships that could be construed as a potential conflict of interest.

## Publisher’s Note

All claims expressed in this article are solely those of the authors and do not necessarily represent those of their affiliated organizations, or those of the publisher, the editors and the reviewers. Any product that may be evaluated in this article, or claim that may be made by its manufacturer, is not guaranteed or endorsed by the publisher.
